# Exploring potential for occupational therapy practice models within areas of social deprivation: A qualitative inquiry within a community-centred food cooperative

**DOI:** 10.1177/03080226221092689

**Published:** 2022-05-15

**Authors:** Richard Adams, Helen Atkin, Richard Lee, Simon S Hackett, Katie L Hackett

**Affiliations:** 1 Department of Social Work Education and Community Wellbeing, 135031 Northumbria University, Newcastle upon Tyne, UK; 2 Population Health Sciences Institute, 5994 Newcastle University, Newcastle upon Tyne, UK

**Keywords:** Collective occupation, foodbanks, occupational injustice, qualitative, health inequalities

## Abstract

**Background:**

A health inequalities gap exists between wealthy and deprived areas. Community-level occupation-focused interventions may support citizens and address inequities within their environments. Since the global financial crash of 2008 and fiscal policy changes within the United Kingdom, there has been a rise in food insecurity. Community volunteer initiatives have responded by providing for their residents. The aim of this study was to explore how occupational therapists may be agents for social change through exploring perspectives of members and volunteers from a community food cooperative in an area of social deprivation.

**Methods:**

Eight semi-structured interviews were conducted with cooperative members and volunteers within a food cooperative established to address food insecurity in a local community. Data were analysed using thematic analysis.

**Results:**

We found three main themes: *It’s Not a Foodbank*, *Shared Hardship* and a cross-cutting theme of *Community*. The results suggest occupation-focused responses can support the development of community and collective occupations thereby contributing solutions to shared problems.

**Conclusion:**

A community-centred, rights-based approach has supported local community need where socio-economic disadvantage and health inequalities were identified. Scope exists for occupational therapists to work genuinely with (not for) communities to address occupational injustice through collective occupation.

## Introduction

### Socio-economic context and health inequalities in the United Kingdom

The global financial crisis of 2008 and a subsequent decade of fiscal measures in the United Kingdom (UK), referred to widely as a policy of austerity, altered the rights, freedoms and incomes of many of the UK citizens experiencing vulnerable circumstances (OHCHR, 2012). Such differentiated impacts have been exacerbated by the COVID-19 pandemic which has led to reduced activity in some sectors of the economy and resulted in an increase in unemployment (Ahmed et al., 2020).

In 2012, Universal Credit, a single benefit designed to replace six existing benefits, was legislated by UK government as part of its welfare reforms (UK Government, 2012). It was introduced in 2013 against a background of cuts to public services and some have criticised it for contributing towards rising levels of poverty and homelessness (Cooper and Whyte, 2017). Many remaining public services have been bound by increasing budget cuts and have been viewed as ‘agents of austerity’ – through having to save money – rather than service the needs of their populations (Cooper and Whyte, 2017). Several studies have shown that the most severe effects of austerity have disproportionately affected the poorest in society (Oxfam, 2013). One of the most reported consequences has been the widening of inequality, particularly health inequality (Marmot, 2020).

### Agents of social control or agents of social change? An occupation-focused response

Participation in meaningful occupation has been held up as a human right (World Federation of Occupational Therapists, 2019). The literature engages with this in two main ways. Firstly, by considering health economics and demonstrating how meaningful occupation may contribute to a cost saving and service cutting narrative of neoliberal austerity (Green and Lambert, 2016). The second response is more politically conscious and critical, embracing concepts of occupational apartheid, (in)justice, deprivation and rights in working with communities (Lauckner et al., 2019; Whalley Hammell and Beagan, 2017), particularly those that are marginalised.

Working with populations in vulnerable circumstances means resolving a fundamental tension between being agents of social change, supporting growth and development, or agents of social control, encouraging adaptation to the prevailing environment (Creek and Cook, 2017). Van Bruggen (2014) concurs, arguing new roles in partnership with communities should be created to address structural inequities while Townsend (2007: 154) refers to ‘just and inclusive societies’ as the kind of social change occupational therapists work towards. Despite the wealth of literature on these injustices, there is very little research into practical responses to them (Galvin et al., 2011). Collective occupations are important in bringing about social transformation (Laliberte Rudman et al., 2019) and further research is required to explore how occupation-focused practitioners, including occupational therapists, can be more socially responsive (Malfitano et al., 2016).

### Community-centred practice

Hyett et al.’s (2019) community-centred practice framework enables occupational therapists to conceptualise engagement with communities. This includes notions of community identity, community occupations, community resources/barriers and processes supporting community participation enablement. In community-centred practice, the community itself is the client, and work is done with, and not for, the community (Hyett et al., 2019). This principle of co-production and co-creation requires critical reflexivity to recognise and address the impact of professional privilege and power (Whalley Hammell, 2013) on community relations and practices.

### Food poverty in the UK

Foodbanks provide emergency food parcels to those in need, often on a referral basis. However, they are politically controversial with widespread use being praised for demonstrating the community-minded nature of society, whilst also receiving criticism for assuaging guilt rather than prompting active engagement – allowing the state to retreat whilst a ‘sticking plaster’ is applied over failures in income levels, job security and welfare support (Williams et al., 2016).

There has been increase in the use of foodbanks in the UK, from 26,000 users in 2008/9 to 1.9 million in 2019/20 (Clark, 2020), and other forms of food welfare with 170 medical experts claiming that food poverty in the UK amounts to a public health emergency (Ashton et al., 2014). The United Nations and Human Rights Watch have declared the inequality in Britain an abuse of human rights (Human-Rights-Watch, 2019; OHCHR, 2012) with its effects linked to a crisis in mental health (Wickham et al., 2020). The Policy Press proposed that despite neoliberalism’s appeals to personal responsibility, the reasons for food poverty and people using foodbanks are structural in their nature, not down to individual characteristics or abilities (Garthwaite, 2016). Food cooperatives are different to food banks. Food cooperatives are food distribution outlets which often offer a choice of food and household items at low cost to its members, giving members choice and autonomy.

The aim of this qualitative enquiry was to explore perspectives of members and volunteers from a community food cooperative in a socially disadvantaged community with a view to illuminating potential for occupation and justice focused social change within communities experiencing social deprivation.

## Methods

### Qualitative approach and research paradigm

A qualitative inquiry was conducted with data being collected through semi-structured interviews. A phenomenological approach was used to understand the stakeholders’ experiences in their own terms. Phenomenology places the person and their experience at the centre of the research (Finlay and Ballinger, 2006). From an ontological perspective, we operated from a critical realist position that gives primacy to the lived experience of the participants that is rooted in a very specific context (Braun and Clarke, 2013). It is the reality of the participants that this study aims to elucidate, with the acknowledgment that this reality might be shared by others in similar settings. For this reason, the epistemological paradigm for this study is contextualism (Braun and Clarke, 2013). The participants’ contexts being crucial to the formation of their own experience and any gained knowledge is local, situated and provisional to that or similar contexts (Tebes, 2005).

### Researcher characteristics and reflexivity

Reflexivity within the study design was maintained by the primary researcher RA (an occupational therapy student) who kept a research journal with the contents being discussed in regular meetings with the supervisory author (KLH), an academic occupational therapist. One example discussion was when the primary researcher (RA) discussed his feelings of being ‘an outsider’ after being stopped in the street by a group of young people on his way to the cooperative. In this moment, he realised that he was seen as a curiosity to them as someone who did not live on the estate. During the supervision session that followed this was discussed, particularly in relation to a subtheme which arose from the data, to try as much as possible to not allow any feelings that arose from that encounter to influence the coding and data analysis and so avoid ‘unconscious editing’ (Berger, 2015).

### Context

The food cooperative is within a housing estate in a city in the Northeast of England. It was established and is managed by a church group using rented premises, adjacent to estate’s housing association offices. It includes a small shop and space for members to have a hot drink and conversation with each other and the volunteers. The food cooperative was established to support residents experiencing food and financial insecurity and to build community. Membership is exclusive to anyone who resides, works or has children attending school within the estate’s postcode area. To join, members are required to pay a small one-off joining fee of £2 and add money to an account, which is then converted to points. Points are used to purchase items from the shop. Refreshments are available free of charge to members. Partly stocked by donations from the church and local businesses, items are available at least 50% cheaper than supermarkets with proceeds going back into the cooperative. The cooperative is part of a network of services run by both the voluntary sector and council. Occupational therapists were not involved in the set-up or running of the food cooperative.

### Sample strategy and recruitment

We sought to gain insights from a cross section of adult participants (aged 18 and over) who were members, volunteers or both members and volunteers of the food cooperative. We used convenience sampling to recruit participants: The cooperative managers informed members as they arrived that a study was being conducted on site and then the researcher approached members in person to explain more about the project with the participant information sheet. An element of snowballing took place as some of the participants encouraged their friends to take part. A total of eight participants were recruited. Participants consisted of six women and two men who were either food cooperative members (*n*=5), food cooperative members who also spent some of their time volunteering at the project (*n*=2), or volunteers who were not users of the food cooperative (*n*=1).

### Ethical approval

Ethical approval for this study was granted from Northumbria University Ethics Committee (ref. 11240) in January 2019. Informed consent was gained via a signed and dated consent form which had been shared with the participant, along with the study participant information sheet prior to any interviews taking place.

### Data collection

Each participant was interviewed once by (RA) during February and March 2019 in a private room at the cooperative, using pre-designed interview schedule (Pope and Mays, 2006) (see Table 1). Interviews were audio recorded and each one lasted between 30 and 90 min.

**Table 1.** Interview schedule.

Cooperative members
1. How would you describe the pantry to someone who didn’t know what it was?
2. How often do you use the pantry?
3. Why do you use the pantry?
4. Have you used any other service similar to the pantry?
5. How has the pantry made a difference to you?
6. Have you noticed any changes within yourself since using the pantry? If so, how?
7. How important is the place/space where the pantry is?
8. What do you think the word ‘community’ means? How would you describe your community?
9. What role do you think the pantry plays in your community?
10. Has the pantry changed the way you think/feel about your community?
11. Why do you think the pantry was set up?
*Volunteers*
1. How would you describe the pantry to someone who didn’t know what it was?
2. How has the pantry made a difference to you?
3. Have you noticed any changes within yourself since using the pantry? If so, how?
4. What motivates you to get involved with the pantry?
5. Why do you think the pantry is important?
6. How important is the place/space where the pantry is?
7. What role do you think the pantry plays in your community?
8. Has the pantry changed the way you think/feel about your community?
9. How would you describe the people who use the pantry?
10. How would you describe the community that the pantry serves?

### Participants and interviews

Table 2 provides information about the participants.

**Table 2.** Demographics of participants.

Participant	Cooperative member (CM) or volunteer (V)	Employment status	Dependents	Lives on the Estate	Gender
1	CM	Employed – zero hours contract	Y	Y	F
2	CM	Universal Credit	Y	Y	F
3	CM	Universal Credit	N	Y	F
4	CM & V	Benefits*	N	Y	F
5	CM & V	Benefits – PIP	N	Y	M
6	V	Employed	N/A	N	F
7	CM	Benefits – PIP	N	Y	F
8	CM	Employed – part time	N	Y	M

*not specified; PIP – Personal Independence Payments.

### Data processing and analysis

Recorded transcripts of the data were transcribed by the first author (RA). Descriptive phenomenology provided a framework for thematic analysis to identify, analyse and report patterns in the data (Braun and Clarke, 2013). This was done following the phases proposed by Braun and Clarke (2013). First, a member of the research team (RA) familiarised themselves with the data before identifying initial codes which were then grouped into themes. The codes and themes were discussed in regular meetings with (KLH). These themes were then analysed in relation to the whole data set. Analysis was inductive to allow the stories of the participants to take precedence (Azungah, 2018). An interpretative element was also applied when using Hyett’s et al.’s (2019) community-centred practice framework as a lens through which to view the impact of the food cooperative.

## Results

### Main themes

**Figure 1.**
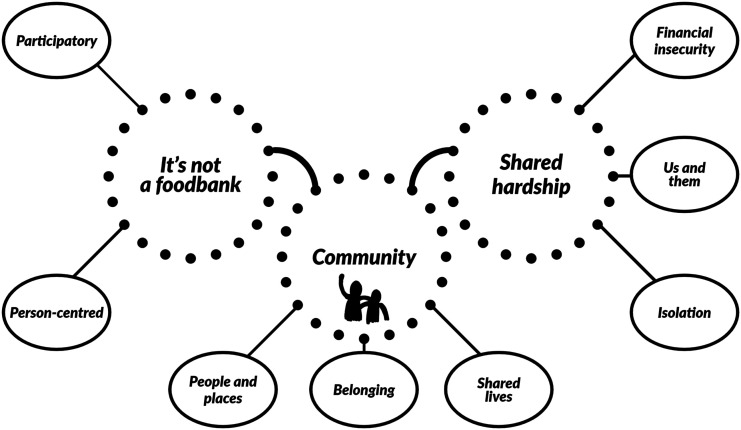
Themes and sub-themes.

Two main themes, with sub-themes, were extrapolated from the data collected: ‘It’s not a foodbank’ and ‘Shared hardship’, with a further third cross-cutting theme which informed our understanding of the data – ‘Community’ (see Figure 1). Notions of community (what it means, who is included and who is not, and how they are maintained) are central to this study which positions communities, rather than just the individuals within them, as the focus of a church-led initiative. Therefore, the cross-cutting theme of community is discussed first to understand and frame the importance and role of the cooperative to the participants. All participants discussed the concept of community during their interviews and how the cooperative related to it. Therefore, by collating and examining those comments first, we hope to contextualise the comments about the cooperative.

### Community

All participants were asked about community to provide context to their responses about the impact of the food cooperative upon their community.

### People and places

The main sub-themes arising from these responses involved the centrality of familiar people and places:

Community for me, I would say that it is…erm…getting to know people…being able to talk, you know … saying hi on the street, looking out for each other (P.2).

As well as the sharing of lives through shared experiences and activities:

My community would be, like, family orientated. Anyone like children, adults, who are struggling, they’re on, maybe, not the best wages or, I mean, I’m on a zero hours contract so, the community, for someone who is in that situation like me, it means we can all raise kids as well as working, and not feel like we have to struggle (P.1).

### Belonging

There was also the sense of belonging resulting from this:

I think you need to be strong and grow trust in a community, and know that it’s a safe place to go…because I think a lot of people in [name of estate] might be scared to go out the door, but if they came in here they’d know that this is a safe community to be in and that it might make them more confident and think, ‘It’s okay this place’ (P.3).

In relation to the cooperative, all participants referred to the positive impact it has had on the local community: ‘the community has always been there but [name of cooperative]… it’s like a foundation, it’s like glued wuh [us] together’ (P.2).

Volunteers at the food cooperative were clear that contributing to the community, promoted a collected sense of identity: ‘we are much more than what we sell’ (P.6). The opportunities the cooperative afforded for residents, including affordable food items and volunteer roles, made the cooperative accessible to anyone living within the estate. With this system, based on locality, and the participatory ethos of the cooperative model, members know they won’t be turned away and have a place where they too can belong.

### Shared lives

Rather than the anonymity of a large foodbank, the cooperative forms relationships with its members and so can offer help and support outside of its immediate remit: ‘if you need anything, they can put a notice around and ask, if you need plates or cups and they’ll try and sort you out, you just leave it with her [the manager]’ (P.4). Mentioned across the interviews was the way the cooperative brought people together: ‘Before you could walk down the street and not know anyone but now, I can walk past people and say, “Hello, oh, you’ve been to [name of the cooperative], haven’t you?’ (P.1). It had provided a starting point for a conversation while being out and about. By providing a space at regular times each week, the cooperative is helping to form social bonds that otherwise would not have existed, with beneficial effects on the wellbeing of members, including the opportunity to relax and feel more at ease:

Yeah, it’s like every Tuesday and Thursday you can come in and…even if you’ve had a really bad week and you can just feel yourself…the weight lifting off your shoulders, even if it’s just having a chat with someone in there for 20 mins you can just feel yourself going ‘Ahhhh!’ (P.2).

The cross-generational appeal of the cooperative was expressed unanimously with participants referring to its popularity for all age groups, especially families and the elderly: ‘I think in this place, especially for the older people, the adults and the mams and dads this is a community in itself because it’s like our little community’ (P.3).

All participants said they wanted to see the cooperative get bigger and/or expand into other areas so that it could help more people: ‘At the moment it’s just in this area but if we could get people from other areas or even go to their area and try to open one there…I think a lot of people would benefit from it’ (P.2).

Since its inception, the food cooperative has grown organically through, predominantly, word-of-mouth recommendations, demonstrating its valued place amongst members. Having local volunteers also bolsters the community-feel of the cooperative and this was a major plus for the participants: ‘you need people who live on the estate who can talk like normal people, and they understand, and they can get people in and this has gotta be a word-of-mouth thing’ (P.3). The way the cooperative is viewed as part of the community is key to both its popularity and perceived importance.

### Shared hardship

A key theme arising from participant responses, was a shared experience of hardship. The three sub-themes, illustrating the kind of shared hardship experienced by the community are financial insecurity, a sense of isolation and an ‘us and them’ mentality.

### Financial insecurity

Several participants mentioned the pitfalls of low pay and/or precarious work such as zero hour’s contracts: ‘you know most people are only one pay-check away from homelessness’ (P.3). Even those in work noticed others in the workplace with more senior roles (and therefore – receiving more pay) also find themselves in a precarious position. One participant spoke of their colleague who had made use of the cooperative: ‘Well, at work a while ago it was me boss who said “I’m on me butt skint” and she went and spent about two quid and came out with 3 full bags of shopping’ (P.7). In addition to this, Universal Credit was singled out across many responses as a major contributor to the struggles local people face: ‘Universal Credit has done for a lot of people’ (P.5). The lumping together of separate benefits into one monthly payment has caused significant problems for people without experience of managing their money in this way. ‘Universal Credit put your benefits all in one and nobody knows where the hell they are’ (P.5). Furthermore, the tightening of rules around Personal Independence Payments (PIP) has led to more insecurity for those living with illness or disability: ‘He’s had problems with his Universal Credit, they’ve stopped his PIP and he had nothing’ (P.8). The main outcome of this has been food poverty ‘they can afford to pay the rent, but they can’t afford to eat…so they’re paying one bit, they’ve got a roof over their heads but what are they going to do about food’ (P.3). Every cooperative member participant mentioned either first or second-hand accounts of running out of food and/or money. Families felt the strain particularly: ‘You’ve got kids you’ve gotta look after them and you’ve gotta have money on your electric, money on your gas, to use your cooker to cook your food, so you know’ (P.2). In this environment, the food cooperative has become indispensable to many in the community: ‘I think…people, especially the ones that come here, would be lost without [name of the cooperative]’ (P.2). While the most obvious advantage it provides its members is cheap groceries, its points system also allows people to load money on to their account when they have it, which helps with budgeting and food security.

### Isolation

Social isolation was cited as key feature of life in a marginal setting. The issue is especially pronounced for the elderly who were described as having few options for social interaction: ‘My grandma was never alone because she had family around her and had lots of friends, but you do get people that are…they’re really lonely’ (P.2). This appears to have led to a generational divide, as young people with few opportunities for meaningful activity become involved in anti-social behaviour and older people, reluctant to go out because of the perceived threat, stay indoors: ‘there’s a lot of older people who don’t get out as much, and there’s all the kids as well. You see them mooching about all over the place and you don’t always want to go out’ (P.7). The withdrawal of local services for young people and adults has compounded the problems of isolation: ‘But there’s nothing on it, nothing for them to do, not even football nets… they closed that police station down – the nearest one is town’ (P.8).

### Us and them

The breakdown of trust between residents and their local institutions and between residents themselves has led to the formation of an ‘us and them’ mentality. Common across the interviews was a negative view of how the estate and its residents are perceived by outsiders. The area’s negative reputation was mentioned by all participants and is a key feature of how they understand their community and its place in the dominant social order. These perceptions are often reinforced by the media: ‘on the news and stuff they say…and you think, “Why?”…You’re making us sound like a warzone’ (P.2). The estate itself also seemed to be especially prone to this mentality on account of its architectural design separating it from the rest of the city: ‘I mean, this place is a bit of an enclave, you know the way it’s been designed and that – everything…is cut off’ (P.8). However, these perceptions can come from residents themselves, directed towards those on the margins of the estate and go some way towards explaining community divisions mentioned across the interviews – between old and young; employed and unemployed; those who are still receiving benefits payments and those who have had them cut; those who have lived here for a while and newer, more transient residents; or those with drug and alcohol problems and those without.

Most participants referred to judgements within the community about being known as someone who was in need or deemed by others as not being able to look after themselves. This contributed to a sense of shame for those who needed to seek help. When asked about where the perceived sense of shame comes from, one interviewee replied:

That actually comes from the community, so it’s the people you live around, in a way…And you know the gossip mongers asking what she’s doing in there an…or there’s a police car out in the street…curtain twitchers…cos you know, as I say, there is a big drug problem (P.3).

Shame and stigmatisation were revealed to be a pervasive part of life in this community. Ideas of being deserving or undeserving permeated discussions of shame:

But then you get people who abuse the system, which is some of the hard things that I find. I had to sit down with the vicar that used to be my line manager and ask ‘How do you differentiate? How do you work out who’s genuine and who’s not?’ and when you see people coming back week after week asking for food vouchers and then you see them walking around in designer gear, with an up-to-date smart phone, smoking proper cigarettes (P.3).

Over time, the removal of services also seems to have led to a divided community on the estate – ‘I mean you’ve got various parts of [the estate] – you’ve got the top end, you’ve got us at the bottom end, you’ve got them on this side, you’ve got them on that side and they never really mix’ (P.3) – suggesting different parts of the community are either reluctant or too intimidated to engage with each other.

### It’s not a foodbank

The theme ‘It’s not a foodbank’ originated from participants. It includes positive attributions towards being a member of a food cooperative such as feeling empowered through buying food and making a contribution rather than receiving it for free from a food bank. The cooperative model enabled its members to participate in a shared community where there were ‘familiar faces’. Further participation was enabled through volunteering opportunities, supporting feelings of empowerment and shared support for a collective project. Participants felt that they had more choice about the types of food they could select for themselves and their families from the cooperative. The cooperative model was also seen to be more sustainable than a foodbank with its members not being dependent on food vouchers that may be issued in limited numbers. Participants experienced the food cooperative as being person-centred, feeling themselves to be valued and important, which promoted a sense of wellbeing through developing trusting relationships and self-confidence. Alongside receiving personalised support within the food cooperative, which led to employment opportunities for some, members felt enabled to participate in wider community activities with their families with funds that they no longer had to set aside for food provision.

The use of foodbanks was expressed in negative terms across all interviewees with higher value being placed upon the resource being set up as a community food cooperative. Being a member of a cooperative through making a contribution helped people to avoid strong feelings of shame, disempowerment and negative characterisations like ‘scrounging’ (P.2). Negative experiences reported by participants related to foodbanks were expressed in terms of shame: ‘I think there’s that shame going to a foodbank’ (P.1); disempowerment: ‘in some ways you feel bad because you’ve got to go cap in hand to someone and say I’ve got nothing, I need something’ (P.2); and frustration ‘they had two carrier bags of food that you couldn’t even eat, I couldn’t eat it’ (P.4). A foodbank was not considered as sustainable: ‘If you’re only allowed 3 foodbank vouchers, how else are you going to feed yourself?’ (P.3). Much of the praise for the food cooperative was initially expressed by participants in terms of its difference to a foodbank. The following sub-themes highlight further benefits of the food cooperative expressed by both service users and volunteers.

### Participatory

The participatory aspect of the cooperative was acknowledged by all participants. Although food can be obtained for free at a foodbank, the participants were positive about the food cooperative as their contributions eradicated potential feelings of shame from relying on a foodbank: ‘you’re not just scrounging as some people would say’ (P.2). They also helped to foster a sense of empowerment over their own lives: ‘it’s giving them that little bit more control and making them feel that little bit better because they are still actually buying it’ (P.3); and removed the frustration that comes with lack of choice over such an important part of our lives – what we choose to put in our and our families’ bodies: ‘I can actually feed myself and my kids’ (P.1). Once a part of the cooperative, members are also able to volunteer and participate more fully in helping to run it. This has powerful effects for the individuals whose wellbeing is improved by regularly lending time and skills to a collective project, as well as for the cooperative itself, where members benefit from seeing familiar faces from within the community, who understand its idiosyncrasies and are invested in it.

### Person-centred

Prominent across participant responses was the person-centred approach of the food cooperative. All participants mentioned the warmth of the environment created by the friendliness of the volunteers, many of whom are local community members and some discussed how it made them feel valued as people: ‘You’re always made to feel that it’s really important what you’re talking about even, it might not be, but to you it really is, and they make you feel like it is important and you’re important’ (P.2). Notable across all interviews was the positive impact on mental wellbeing a visit to the cooperative left members with: ‘Just coming in here for 20 min…it’s like, it does you good…it makes you feel really happy and good…’ (P.1). Personal growth, in particular confidence-building, was one of the most common impacts participants (volunteers and members) reported after regularly using the cooperative: ‘when I first came, I was like, really shy and didn’t really talk to people but now…I’ve kind of come out of me shell meself’ (P.2). Once confidence and trust are established, it provides a foundation for other types of support that members may need such as the development of life skills:

Someone that uses [name of the food cooperative] now, had problems filling forms in so he’d bring the forms in and we’d sit and fill them in, it was getting over that first hurdle and we got him more confident and a bit more confident and then he got a job… you’re getting help with other things that are part of the reason you’re in food poverty in the first place (P.3).

This has further positive benefits as participants reported being able to participate more fully in wider society because of the skills and savings the food cooperative has helped them with:

I come here and I put a fiver on [credit added to their account] and it would be £20 in Morrisons…that’s £15 I could put into the kids Christmas fund or go and get them a treat, even a McDonald’s or…take them to soft play and that is something I wouldn’t have been able to do before cos you haven’t got the money to do it like (P.2).

The combination of a person-centred and a participatory ethos was praised by the participants, all of whom cited the friendly, supportive environment and the ability to have choice and control over their consumption as equally important as the financial savings they made.

## Discussion

This study addresses calls in the occupational therapy and occupational science literature for pragmatic examples of how occupational therapy might respond to structural inequalities that impact on the wellbeing of communities and individuals’ through socially responsive practice (Malfitano et al., 2016), that prioritises the shared experiences of communities not just individuals (Lauckner et al., 2019; Lavalley, 2017). By observing examples such as community-centred food cooperatives, occupational therapists may learn how they could better position themselves to be agents of social change rather than social control.

The findings of the study suggest that the cooperative fulfils an important role for both individuals and the community; not just in relation to food but in terms of their overall wellbeing and ability to participate in meaningful occupations. The food cooperative is a collective, offering a community-centred approach which has had substantive impacts on the rights, freedoms and opportunities of the participants. It has provided a community resource (Hyett et al., 2019), which recognises the diversity of individuals, the strengths of the community while enabling access to affordable food and opportunities to address financial literacy. This has been realised, through support with individual occupations such as budgeting and form completion and the co-occupation of volunteering, leading to members being able to overcome barriers to wider participation in society. This has worked on different levels with some members using the money saved on groceries to participate in ‘luxury’ activities such as soft play or swimming for their children; others making use of their expanded social network to collectively source previously inaccessible items such as homewares; and in some cases, just making use of a safe, welcoming space on a twice weekly basis to help overcome social isolation

Participating in the food cooperative enables its members to collectively overcome some shared problems of social deprivation. It represents a refreshing alternative to foodbanks and community spaces which have been affected by austerity (Williams et al., 2016). In this way, the cooperative, as a meso-level, collective occupation, is clearly contributing to the social fabric and sense of community within the estate (Kantartzis and Molineux, 2017). As others have noted, individuals mirror the values encoded into their environments (Verhaeghe and Hedley-Prôle, 2014). A change to that environment can lead to a change in individuals and the communities that shape them. The participants suggest that that change is happening in the transactional way (Lavalley, 2017) as their individual development mirrors the development of the community around the cooperative. By involving volunteers from that community, the cooperative is becoming embedded so that it is both for, and of the community. This is what Lauckner et al. describe as a key distinction between community-centred practice, like the cooperative, and community-based practice which involves communities being done to rather than with (Lauckner et al., 2019) and is one of the key principles of participation enablement (Hyett et al., 2019).

This research has indicated that to succeed in community-centred practice, practitioners must genuinely work *with* communities. It suggests that engagement with issues of food poverty through food cooperatives is one way in which occupational therapists can address social and occupational inequalities and direct their actions towards a socially orientated, collectivist occupational therapy (Malfitano and Lopes, 2018). Such actions might include identifying collective approaches to addressing financial literacy, supporting access to nourishing meals, volunteering and on a wider scale – facilitating intergenerational dialogue with local government to address wider community occupational injustices.

### Limitations

The small sample size and locality focused inquiry limits the transferability of this study; however, the study was designed to give space to the detailed experiences of each participant. The sampling process was also convenience-based. Purposive sampling could have resulted in a more diverse and representative sample with richer data (Braun and Clarke, 2013). A pragmatic approach was taken, due to the difficulty of arranging interviews in advance and the fact that access to the food cooperative and its members was only possible on its two regular opening days. Research of additional community-centred initiatives could further expand understanding of how occupational therapists may work with communities to improve participation in meaningful occupations by collectively addressing collective issues affecting whole communities. Examples may include groups who collectively experience social isolation and loss of confidence following the COVID-19 pandemic.

## Conclusion

In this study, we aimed to answer calls in the literature for socially responsive practice that prioritise the shared experiences of communities, not just individuals. Occupational therapists should consider how they may work *with* (not for) communities experiencing social deprivation, be agents of social change by engagement and co-facilitate a collective approach to address identified occupational injustices. This might be through supporting skills development, facilitating access to affordable and nourishing food and creating opportunities for connection and belonging through engagement in meaningful occupations which support health and wellbeing and positively impact on the rights, freedoms and opportunities of community members.

## Key Findings

• To succeed in community-centred practice in areas with structural inequalities, practitioners must genuinely work *with* communities.
• Working with communities, occupational therapists can facilitate meaningful occupation and autonomy.

## What the study has added

This study has identified some practical insights in how occupation-focused practitioners might embrace concepts of occupational apartheid, (in)justice, deprivation and rights by working with a community in a marginal setting.
